# Transapical intramyocardial septal microwave ablation in treatment of hypertrophic obstructive cardiomyopathy: 12-month outcomes of a swine model

**DOI:** 10.1186/s13019-024-02677-z

**Published:** 2024-04-13

**Authors:** Mi Zhou, Zhaolong Li, Yun Liu, Yuehua Fang, Le Qin, Wenjie Yang, Fuhua Yan, Qiang Zhao

**Affiliations:** 1 Department of Cardiovascular Surgery, Ruijin Hospital, Shanghai Jiao Tong University School of Medicine, Shanghai, 200025 China; 2 Department of Cardiology, Ruijin Hospital, Shanghai Jiao Tong University School of Medicine, Shanghai, 200025 China; 3 Department of Radiology, Ruijin Hospital, Shanghai Jiao Tong University School of Medicine, Shanghai, 200025 China

**Keywords:** Transapical septal microwave ablation, HOCM, Animal experiment

## Abstract

**Background:**

To date, the extended Morrow procedure is considered the gold standard treatment for patients with obstructive hypertrophic cardiomyopathy who experience severe symptoms and are unresponsive to medication treatment. We therefore aimed to perform transapical intramyocardial septal microwave ablation to reduce the thickness of the interventricular septum myocardium in a minimally invasive method.

**Methods:**

Fourteen swine were divided to form either a microwave ablation group (*n* = 7) or a sham group (*n* = 7). In the microwave ablation group, a transapical microwave antenna was inserted into the septum to ablate each myocardial segment at 40 W for 1 min, while in the sham group, the same operation was performed but without power output. We used echocardiography, electrocardiogram, during the operation. And added computerized tomography, cardiac nuclear magnetic resonance during follow-up.

**Results:**

Segment hypokinesis was observed in all swine immediately following ablation. Compared with the sham group, the thickness of ablated segments in the ablation group decreased significantly 1 month post-operation (ablation group, 5.53 ± 1.00 mm vs. 8.03 ± 1.15 mm, respectively, *P* < 0.01; sham group, 8.40 ± 0.94 mm vs. 8.21 ± 1.09 mm, respectively, *P* = 0.081), and the outcome was still observed 1 year post-operation (ablation group, 3.36 ± 0.85 mm vs. 8.03 ± 1.15 mm, respectively, *P* < 0.01). No perforation of the septum was observed during the procedure or follow-up, and no heart failure or sudden cardiac death occurred during postoperative feeding.

**Conclusions:**

Transapical intramyocardial septal microwave ablation can effectively and safely produce a large region of necrosis. This technique can potentially mimic surgical myectomy while avoiding cardiopulmonary bypass and median sternotomy in high-risk hypertrophic obstructive cardiomyopathy patients.

**Supplementary Information:**

The online version contains supplementary material available at 10.1186/s13019-024-02677-z.

## Background

Hypertrophic cardiomyopathy (HCM) is one of the most common heritable cardiovascular diseases, affecting approximately 1 in 500 individuals [1]. Obstructive hypertrophic cardiomyopathy (HOCM), a specific form of HCM, is accompanied by obstruction of the left ventricular outflow tract (LVOT). Patients with LVOT obstruction experience dyspnea and/or chest pain with exertion, often accompanied by arrhythmia, and have a significantly poor prognosis in terms of life expectancy and quality of life [2]. Surgical septal myectomy, namely, the extended Morrow procedure, is considered to be the gold standard treatment for HOCM patients with severe symptoms that are unresponsive to optimal medication treatment [3, 4]. This surgical procedure can completely relieve the pressure gradient of the LVOT and reduce the associated mitral regurgitation. Most importantly, it provides a satisfactory long-term prognosis [5].

More than 70 extended Morrow procedures are performed at Ruijin Hospital every year, where we routinely resect approximately 5–15 cm [3] of myocardium from each patient, with excellent clinical outcomes. However, cardiopulmonary bypass and median sternotomy delay recovery after surgery. The high risk patient is in the dilemma and it is reasonable to find an alternate treatment. Trying to find a new way to diminish these traumas during the operation, we aim to utilize a swine model in order to perform transapical intramyocardial septal microwave ablation, which can cause extensive necrosis (as with the extended Morrow procedure), through a mini-intercostal incision with echocardiography guidance.

## Methods

All experiments were performed in accordance with protocols approved by the Committee for Animal Research of Ruijin Hospital affiliated with Shanghai Jiao Tong University and complied with the 2011 “Guide for the Care and Use of Laboratory Animals.” All animal experiments were performed at the Yinshe Experimental Animal Center, Shanghai. The microwave ablation equipment (supplement matertal) was provided by the Vison-China Medical Devices R&D Center (Nanjing, China).

### Microwave ablation on swine hearts in vitro

Microwave ablation (Vison-China Medical Devices R&D Center, Nanjing, China) [6, 7] was performed on post-mortem swine hearts at differing power levels for various periods of time. Sixty-five post-mortem swine hearts were divided into 13 groups and myotomy was performed immediately following ablation. By measuring and comparing the scope of necrosis, we determined that ablation at 40 W power output persisting for 1 min resulted in a sufficiently large and stable necrosis scope.

### Microwave ablation on living swine models

Fourteen Chinese swine (6–9 months old) were used in this study and were randomly divided into two groups: the Microwave Ablation (MA) group (*n* = 7) and the sham group (*n* = 7). All swine underwent anesthetic induction with an intramuscular injection of xylazine hydrochloride (0.025 ml/kg), and inhalation anesthesia with orotracheal intubation and a heart monitor was used during surgery. Fentanyl (2 µg/kg) was administered to control pain and intravenous glucose and saline solutions were continuously infused throughout the procedure.

**Fig. 1:**
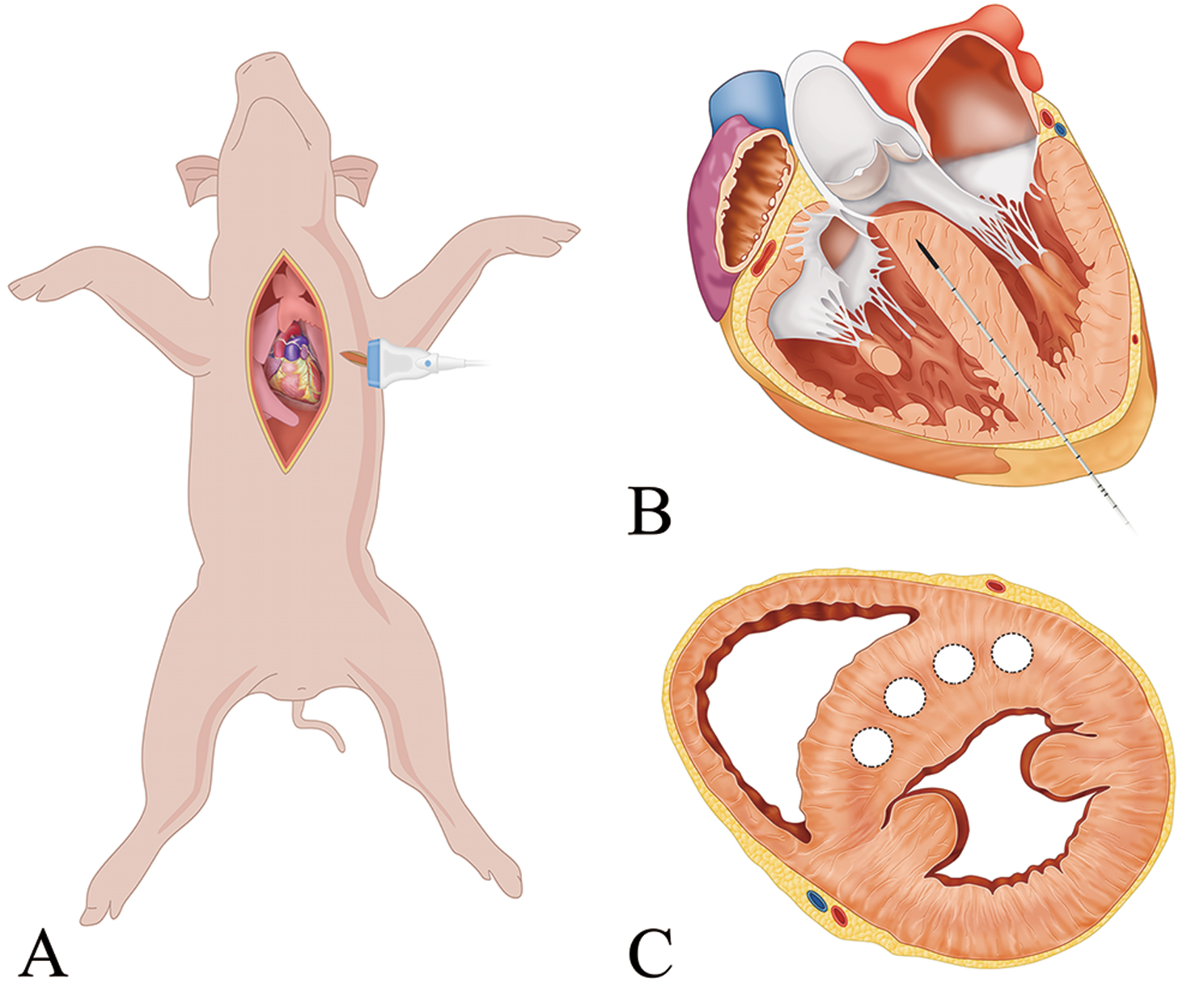
Diagram showing microwave ablation on living swine model. (**A**) Swine were laid in the supine position. The chest was opened and a left anterolateral incision in the fifth intercostal was made for the ultrasonic probe. (**B**) The microwave antennas were inserted via the apex into the interventricular septum under the guide of echocardiography. (**C**) Several antennae were used to produce an extensive ablation. The four white dotted circles showed four ablation regions in one short-axis view

The swine were placed in the supine position with their limbs fixed. For this experiment, the chest was opened with a standard median sternotomy as the cardiac apex of the swine was behind the sternum, and the transesophageal echocardiography was substituted by the epicardial echocardiography. To determine the feasibility of epicardial echocardiography, a left anterolateral incision was made in the fifth intercostal space for the ultrasonic probe. A suture was placed in the posterior pericardium opposite the oblique sinus to facilitate exposure of the apex. The microwave antenna was inserted transapically into the interventricular septum under epicardial echocardiography guidance. The antenna was positioned in the middle of the basal interventricular septum, approximately 10 mm away from the aortic valve and membranous septum (Fig. 1). Ablation was achieved with 40 W power output persisting for 1 min (Position 1, P1). We completed a second ablation after drawing the antenna back by 15 mm (Position 2, P2). The left ventricular anterior wall and posterior septum could also be extended ablated using different puncture angles. In the sham group, the microwave antenna was inserted at the same position as the interventricular septum without output power (supplement material).

### Echocardiography and EKG

Echocardiography was performed using a GE Vivid E9 instrument (GE Healthcare). The amplitude of the ventricular wall motion was observed using the M-mode. Two-dimensional echocardiography was used to measure wall thickness and left ventricular ejection fraction (LVEF). Baseline and follow-up data were collected using transthoracic echocardiography (TTE) and data were collected using epicardial echocardiography during the operation. The EKG was recorded for every swine prior to the experiment, during the procedure, and at 1 month, 6 months, 9 months and 12 months follow-up time points.

### Cardiac magnetic resonance (CMR) and cardiac CT

We performed CMR (GE Healthcare, Boston, MA, USA) and cardiac CT (SIEMENS, Munich, Germany) 3 and 12 months after the procedure. Late gadolinium-enhanced (LGE) MRI was used to verify formation and location of scar tissue following ablation and cardiac CT was used to detect anatomical changes with high resolution.

### Histological analysis

Twelve months after the procedure, the swine were euthanized with an overdose of sodium pentobarbitone, and their hearts were fixed in formalin (10%). For analysis, tissue was taken from three regions: the ablated area, peri-ablated area, and a normal area. Tissue was treated using alcohol dehydration, xylene treatment, and paraffin embedding. Five-micrometer-thick sections were stained with hematoxylin and eosin (H&E) and Masson’s trichrome. The sections were analyzed using a digital microscope.

### Statistical analyses

Continuous data were compared using a Student’s t-test or analysis of variance and expressed as mean ± standard deviation. Categorical data were compared using the chi-square test and expressed as percentages; all P values were two-sided, and a P value of < 0.05 was considered statistically significant. Statistical analyses were performed using SPSS version 20 (IBM SPSS Inc., Chicago, IL, USA). Dr Zhou has full access to all the data in this study and takes responsibility for its integrity and the data analysis.

## Results

### Ablation results on post-mortem swine hearts

The scope of microwave ablation on postmortem swine hearts with different power outputs and time periods are listed in Table 1. We determined that ablation at 40 W persisting for 1 min produced a suitable (mean) and steady (variance) necrosis scope. Therefore, we selected this strategy for use throughout the remaining swine experiments.

**Table 1:** Experimental outcomes for microwave ablation on postmortem swine hearts

Power Output (W)	Time (s)	Length of necrosis (mm) (mean ± SD)	Width of necrosis (mm) (mean ± SD)
30 W	30	12.0 ± 0.5	7.0 ± 0
	60	13.5 ± 0.5	11.8 ± 0.9
	90	16.5 ± 0.8	15.0 ± 1.0
	120	17.8 ± 0.4	17.3 ± 1.3
40 W	10	0 ± 0	0 ± 0
	15	11.2 ± 0.8	6.8 ± 0.8
	30	15.2 ± 1.0	9.2 ± 1.0
	60	17.0 ± 0	14.2 ± 0.2
	90	17.2 ± 0.2	17.8 ± 1.6
	120	19.2 ± 0.9	19.8 ± 0.3
40 W	15, twice	12.6 ± 0.7	8.0 ± 0
	15, thrice	13.0 ± 0.3	9.8 ± 0.3
	15, quartic	15.6 ± 2.3	9.4 ± 0.5

The parameters is showed as mean ± standard deviation

### Results of ablation on swine model

**Fig. 2:**
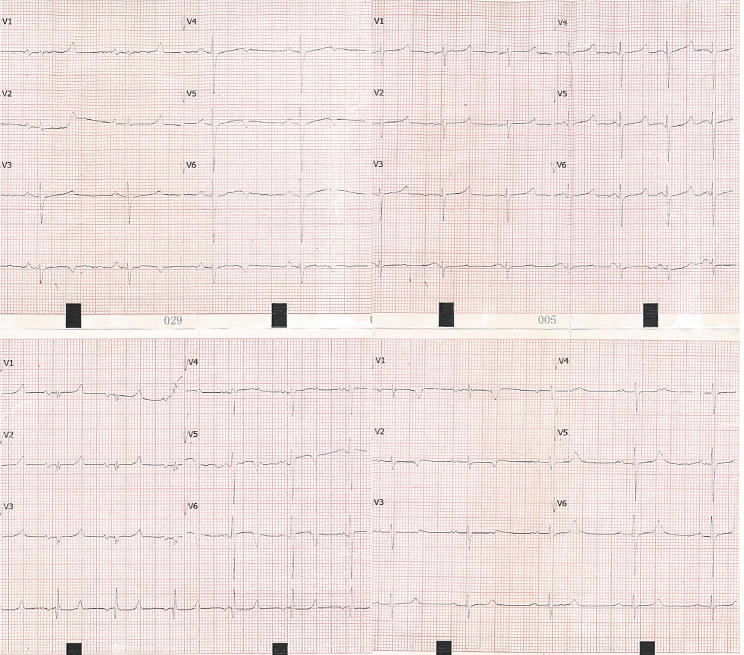
Electrocardiograms 9 months following ablation. Electrocardiograms showing sinus rate of swine from the microwave ablation group 9 months following ablation

Ventricular fibrillation was observed when performing all microwave ablations during the first stage of the preliminary experiment. Eliminating power output and applying electrical defibrillation resulted in the immediate recovery of the sinus rhythm. Additional ablation experiments revealed that ventricular fibrillation could be avoided when systolic blood pressure was maintained above 120 mmHg. Occasionally, ventricular premature beats or paroxysmal ventricular tachycardia were observed during ablation but recovered within 30 s following an intravenous injection of 2% lidocaine hydrochloride. We recorded no rapid ventricular arrhythmia or bundle-branch heart block during follow-up (Fig. 2).

All swine underwent the indicated microwave ablation strategy with no perioperative deaths. Furthermore, they lived for an additional year or more following surgery, with normal athletic ability and appetite. No heart failure, syncope, or sudden cardiac death was observed during postoperative feeding.

### Echocardiographic results

**Fig. 3:**
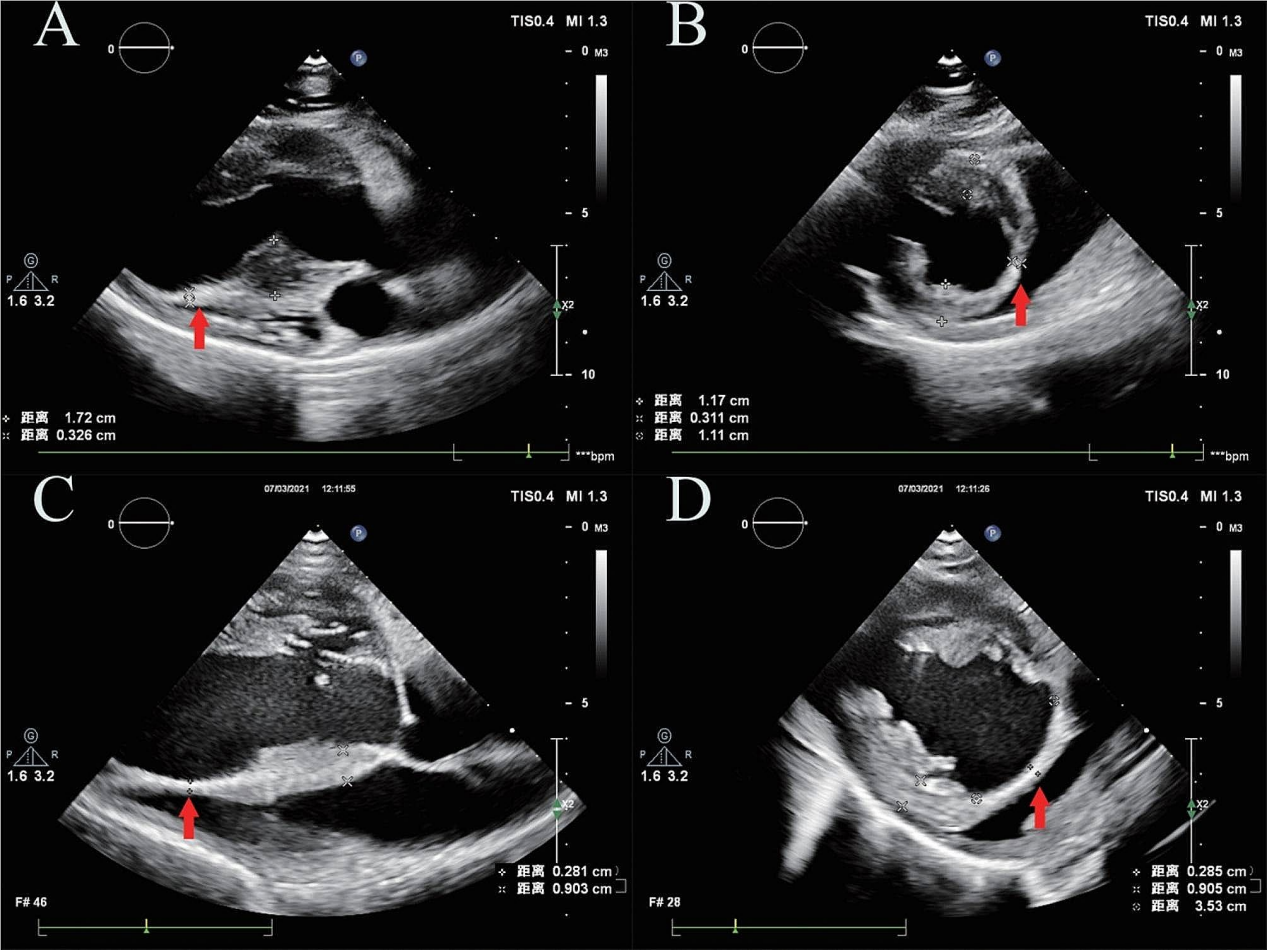
Echocardiography of two swine from the microwave ablation group 1 year following ablation. (**A**) and (**B**) show parasternal long-axis and short-axis views of swine with normal ablation, in which the ablation region was similar to the Morrow procedure. C and D show parasternal long-axis and short-axis views of swine with extensive ablation, in which the ablation region was similar to the extended Morrow procedure. The red arrow indicates the ablated region of the septum, which is hyperechoic and thinner

No perforation of the interventricular septum was observed during the ablation procedure nor during follow-up, and no iatrogenic aortic valve injuries occurred in the MA group. Despite no demonstrated decrease in thickness immediately following the operation, hypokinesis of the ablated segment was immediately observed across all MA swine, but not within the sham group. In addition to this, myocardial contrast echocardiography also demonstrated perfusion defects following the procedure. We ablated two positions (Position 1, P1; Position 2, P2) in each swine. A significant decrease in ablated wall thickness was observed in the MA swine during 1 month of follow-up, while no difference was found at 6 and 12 months post-operation compared with 1 month post-operation (Fig. 3). With regards to the sham group, no change in wall thickness was observed, except in the first ablation position (P1) at 6 months and the second ablation position (P2) at 1 year post operation. However, as the mean values were close, it is possible that the observed differences arose from measurement error or growth and development (Table 2). In addition to this, when follow-up LVEF was compared with baseline or with the sham group, we also determined that microwave ablation immediately affected left ventricular systolic function post-ablation, with LVEF returning to normal within 1 month. The left atrial diameter did not increase after the procedure.

**Table 2:** Thickness of microwave ablation positions (P1 and P2) accessed via echocardiography

Ma	Before MA (mm)	Immediately After MA (mm)	1 Month After MA (mm)	6 Months After MA (mm)	1 Year After MA (mm)
P1	8.03 ± 1.15	9.61 ± 1.54 *P* = 0.01^a^	5.53 ± 1.00 *P* < 0.01^a^	3.34 ± 0.83 *P* < 0.01^a^	3.36 ± 0.85 (41.8%) *P* < 0.01^a^
P2	9.43 ± 0.87	10.94 ± 2.27 *P* = 0.07^a^	5.74 ± 0.96 *P* < 0.01^a^	3.87 ± 0.97 *P* < 0.01^a^	3.76 ± 0.89 (39.9%) *P* < 0.01^a^
LVEF(%)	58.18 ± 5.68	51.77 ± 7.63 *P* = 0.005^b^	57.17 ± 4.50 *P* = 0.462^b^	59.51 ± 5.33 *P* = 0.227^b^	59.38 ± 4.69 *P* = 0.674^b^
**Sham**					
P1	8.21 ± 1.09	8.23 ± 1.07 *P* = 0.36^a^	8.40 ± 0.94 *P* = 0.081^a^	8.67 ± 0.80 *P* = 0.038^a^	8.70 ± 0.74 *P* = 0.041^a^
P2	9.51 ± 0.83	9.51 ± 0.83 *P* = 0.36^a^	9.57 ± 0.94 *P* = 0.618^a^	10.01 ± 1.18 *P* = 0.052^a^	9.91 ± 1.06 *P* = 0.043^a^
LVEF(%)	58.57 ± 7.83 *P* = 0.918^c^	58.28 ± 6.97 *P* = 0.121^c^	58.86 ± 3.85 *P* = 0.466^c^	59.71 ± 5.82 *P* = 0.948^c^	59.56 ± 4.48 *P* = 0.777^c^

Ma: microwave-ablated group; MA: microwave ablation; P1: microwave-ablated position 1; P2: microwave-ablated position 2; sham: sham group. Results expressed as mean ± standard deviation of thickness at the follow-up time. P values of paired t-test of thickness at the follow-up time compared with the thickness prior to ablation. Percentages expressed in parentheses in the final column represent the thickness of the ablated region 1 year after MA divided by the thickness prior to MA. ^a^ indicates paired t-tests of the thickness at follow-up compared with the thickness prior to ablation. ^b^ indicates paired t-tests of the LVEF at follow-up compared with the LVEF prior to ablation. ^c^ indicates t-test of the LVEF in the Ma group compared with the LVEF in the sham group during the same period

### CMR and CT results

**Fig. 4:**
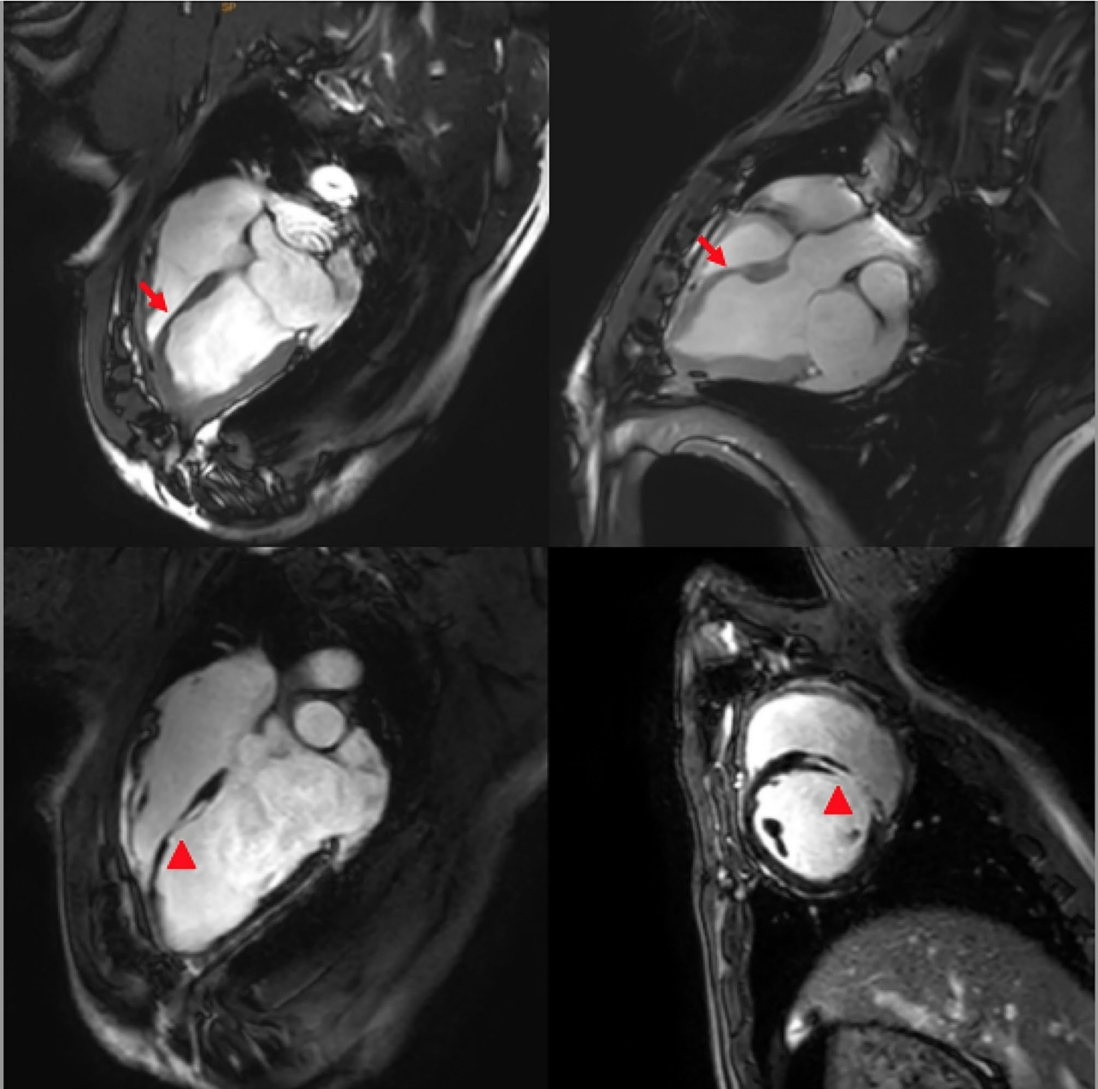
CMR imaging showing a reduction in the thickness of the ablated region. LGE was noted in the mid-wall of the IVS due to fibrosis in the ablated region (red arrows in the superior pictures indicate the thinner ablated region, red triangles in the inferior pictures indicate myocardial fibrosis)

Contrast-enhanced CMR imaging revealed a reduction in the thickness of the ablated segments in both the long- and short-axis slices 6 months and 1 year post operation. LGE was evident in the center of the ablated wall, observed through scar formation (Fig. 4). Furthermore, the CT reconstruction technique demonstrated hypokinesis of the ablated segments (Video 1, Supplementary Material).

### Histology examinations

**Fig. 5:**
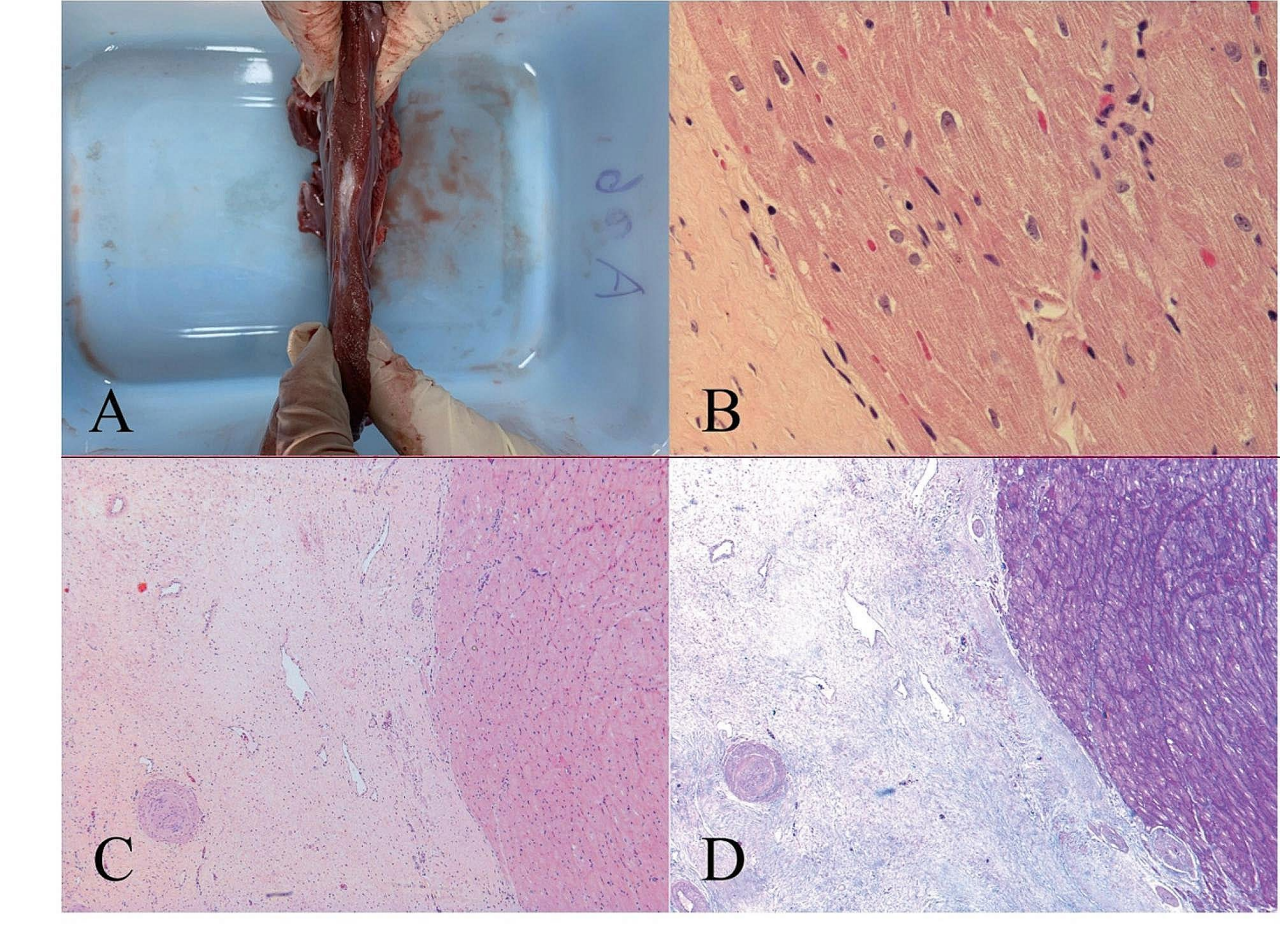
Post-ablation Histological results at 1 year. (**A**) The microwave ablation region (black arrow) was a concave and pale area of infarct with no carbonization and shown to be within the center of the septum with no observable injury to the endocardium. (**B**) A distinct boundary (red arrow) was observable between the ablated region (pink stain) and normal myocardium (red stain) as shown via H&E (magnification ×200). (**C**) The peri-ablated region (red arrow) presented as normal myocardial cells via H&E stain (magnification ×50) (**D**) The ablated area and peri-ablated area (red arrow) are shown via Masson staining used on the same section as those in C. Fibrosis scar tissue is characterized by blue staining (magnification ×50)

Histological examination confirmed that no epicardial coronary arteries were injured during the surgery. Importantly, Fig. 5 shows that the ablated scar region is thinner than the surrounding tissue, and that the borders between the ablated and normal tissues are clear and evident. H&E staining shows the scar tissue is characterized by pink staining with little structures and morphology of spindle cell. In addition to this, Fig. 5 also shows Masson’s trichrome staining section in which the cardiomyocytes (stained red) can be differentiated from fibrosis (stained blue).

## Discussion

According the data of this animal experiment, we projected that transapical microwave ablation can potentially mimic surgical myectomy while avoiding cardiopulmonary bypass and median sternotomy in high-risk hypertrophic obstructive cardiomyopathy patients.

While clinical recognition and management strategies for hypertrophic cardiomyopathy have improved significantly in recent years [8], the extended Morrow procedure is still considered the gold standard treatment for patients with HOCM. Surgical myectomy has shown excellent long-term efficacy and safety, with complete resolution of LVOT gradients and long-term survival approach to demographic-matched populations [9]. Indeed, the 2020 American Heart Association/American College of Cardiology guidelines for HCM suggest that surgical myectomy is reasonable for patients with obstructive HCM who remain severely symptomatic despite guideline-directed management and therapy. Furthermore, these guidelines also recommend alcohol septal ablation (ASA) as a reasonable option for patients with unacceptable surgical risk [10]. However, it should be noted that ASA is associated with a 10–15% rate of complete atrioventricular block and is dependent on the coronary anatomy [11]. Compared with ASA, trans-coronary septal ablation with microspheres has the advantage of confining the microspheres to the vasculature which prevents leakage, and thereby confining necrosis to the region of perfusion. This ultimately poses a decreased threat to the branch of the left bundle [12]. In contrast, radiofrequency (RF) ablation, which is both minimally invasive and independent of the coronary anatomy, has been used for endocardial ablation to reduce the pressure gradient in the left ventricular outflow tract. However, one study reported that the reduction in septal thickness was < 2 mm for the retrograde aortic approach under electroanatomic guidance, with an incidence of pacemaker implantation of 8.8–20% [13]. Recently, the Liwen procedure has provided a novel approach that applies radiofrequency energy through the transseptal approach using echocardiography guidance [14].

While, use of microwave ablation has a number of advantages compared with RF ablation in addition to has several theoretical benefits. As microwaves heat tissue through the agitation of water molecules, they offer a more direct method of heating compared with other ablation energies. Moreover, this form of ablation has greater potential in organs with high blood perfusion as it has minimal heat sink effects near large blood vessels (> 3 mm) [15], and does not require grounding pads, which reduces electrical damage. The potential benefits of microwave technology include consistently higher temperatures, larger ablation areas, improved convection profile, and less procedural pain. Importantly, it also allows for shorter ablation time and is capable of powering several antennas from the same source without switching or bipolar techniques [16]. As such, we were able to insert 3–4 antennas simultaneously to create extensive necrosis within a short time frame (1 min).

Due to the excised hypertrophic myocardium with endocardium, a left anterior branch block is common ($\sim$38.8%), and high-grade atrioventricular block sometimes occurs (< 1%) following myectomy [17] Microwave ablation can aid in the prevention of injuries to the bundle branch by delivering energy to the mid-wall of the interventricular septum. To our knowledge, we are the first team to use microwave ablation to mimic surgical myectomy in the treatment of HOCM. Our approach with 3–4 antennas makes an effective and extensive intramyocardial septum ablation, similar to the scope of surgery. Our findings indicate that microwave ablation was well tolerated by the swine and created precise, large lesions without any observable tissue charring. Moreover, by performing the operation with a mini-thoracotomy, we were able to puncture the apex cordis at different angles and position each antenna in a different segment with the guidance of echocardiography. These results indicate that microwave ablation is able to mimic the scope of excision of the extended Morrow procedure. No obvious postoperative arrhythmias were observed.

Despite our promising results, this study has some limitations. First, we performed this operation on healthy swine, and it is possible for a paradoxical increase in septal thickness during surgery to occur due to tissue edema in patients with HOCM. However, segmental hypokinesis were observed immediately following ablation, and systolic anterior motion of the mitral valve should be stopped. Therefore, similar to ASA, septal reduction could immediately reduce left ventricular outflow tract gradients. Finally, while this procedure continues to decrease the thickness of the ablation segment over the following months, it cannot be used to treat anomalies of the mitral valve apparatus and papillary muscles.

## Conclusion

In conclusion, intramyocardial septal microwave ablation may be a minimally invasive alternative to the extended Morrow procedure as it is a safe and effective method for reducing thickness within the ablated segment. This procedure can be used to ablate several segments of the ventricular septum and can mimic the excision scope of the extended Morrow procedure, without requiring cardiopulmonary bypass or sternotomy. Clinical trials are needed in the future to prove the safety and efficacy of intramyocardial septal microwave ablation in patients with HOCM.

### Electronic supplementary material

Below is the link to the electronic supplementary material.

Supplementary Material 1

Supplementary Material 2

Supplementary Material 3

Supplementary Material 4

## Data Availability

All data generated or analysed during this study are included in this published article [and its supplementary information files].

## Abbreviations

ASA: alcohol septal ablation
CMR: cardiac magnetic resonance
EKG: electrocardiogram
H&E: hematoxylin and eosin
HCM: hypertrophic cardiomyopathy
HOCM: obstructive hypertrophic cardiomyopathy
LGE: late gadolinium enhanced
LVEF: left ventricular ejection fraction
LVOT: left ventricular outflow tract
MA: microwave ablation
RF: radiofrequency
