# Supplementary material for: Transapical intramyocardial septal microwave ablation in treatment of hypertrophic obstructive cardiomyopathy: 12-month outcomes of a swine model
Source: J Cardiothorac Surg. 2024 Apr 13;19:205. doi: 10.1186/s13019-024-02677-z (PMC11015544; doi:10.1186/s13019-024-02677-z)
Supplement: Supplementary file 1 — Supplementary Material 1 [file 13019_2024_2677_MOESM1_ESM.docx]

Supplement Table. 1 Thickness of microwave ablation(MA) positions accessed from echocardiography

| Group | Before MA (mm) | | Immediately After MA (mm) | | 1 Month After MA (mm) | | 6 Months After MA (mm) | | 1 Year After MA (mm) | |
| --- | --- | --- | --- | --- | --- | --- | --- | --- | --- | --- |
|  | MA P1 | MA P2 | MA P1 | MA P2 | MA P1 | MA P2 | MA P1 | MA P2 | MA P1 | MA P2 |
| Ma |  |  |  |  |  |  |  |  |  |  |
| 1 | 7.2 | 8.6 | 10.3 | 12.1 | 5.7 | 5.7 | 3.6 | 3.7 | 3.6 | 3.5 |
| 2 | 9.9 | 10.6 | 11.9 | 13.3 | 7.2 | 7.4 | 4.8 | 5.6 | 4.9 | 5.3 |
| 3 | 6.6 | 8 | 7.9 | 7.6 | 4.6 | 5.1 | 3.1 | 3.3 | 3 | 3.2 |
| 4 | 8.7 | 9.5 | 9.2 | 9.9 | 5.6 | 5.9 | 2.3 | 3.3 | 2.4 | 3.2 |
| 5 | 8.7 | 9.5 | 9.2 | 9.9 | 5 | 4.9 | 2.8 | 2.9 | 2.9 | 2.9 |
| 6 | 7.1 | 9.8 | 7.8 | 9.8 | 4.3 | 4.7 | 2.9 | 3.5 | 2.8 | 3.5 |
| 7 | 8 | 10 | 11 | 14 | 6.3 | 6.5 | 3.9 | 4.8 | 3.9 | 4.7 |
| Sham |  |  |  |  |  |  |  |  |  |  |
| 1 | 9.7 | 9.3 | 9.7 | 9.3 | 9.5 | 9.1 | 9.5 | 9 | 9.5 | 9.2 |
| 2 | 7.8 | 9.8 | 7.8 | 9.8 | 8.1 | 10.3 | 8 | 10.9 | 8.1 | 10.6 |
| 3 | 7.8 | 10 | 7.8 | 10 | 8.1 | 10.3 | 8 | 10 | 8.1 | 9.9 |
| 4 | 8 | 9 | 8 | 9 | 8.5 | 9.2 | 8.7 | 9.4 | 8.6 | 9.3 |
| 5 | 9.6 | 10.5 | 9.6 | 10.5 | 9.8 | 11.7 | 9.9 | 11.5 | 9.9 | 11.4 |
| 6 | 6.6 | 8 | 6.7 | 8 | 7.6 | 8.3 | 7.9 | 8.3 | 8 | 8.3 |
| 7 | 8 | 10 | 8 | 10 | 8.5 | 10.2 | 8.6 | 11 | 8.7 | 10.7 |

Ma: Microwave ablated group; MA: Microwave ablation; P1: Microwave ablated Position 1; P2: Microwave ablated Position 2; Sham: Sham group.
