# Supplementary figures and images for: Transapical intramyocardial septal microwave ablation in treatment of hypertrophic obstructive cardiomyopathy: 12-month outcomes of a swine model
Source: J Cardiothorac Surg. 2024 Apr 13;19:205. doi: 10.1186/s13019-024-02677-z (PMC11015544; doi:10.1186/s13019-024-02677-z)

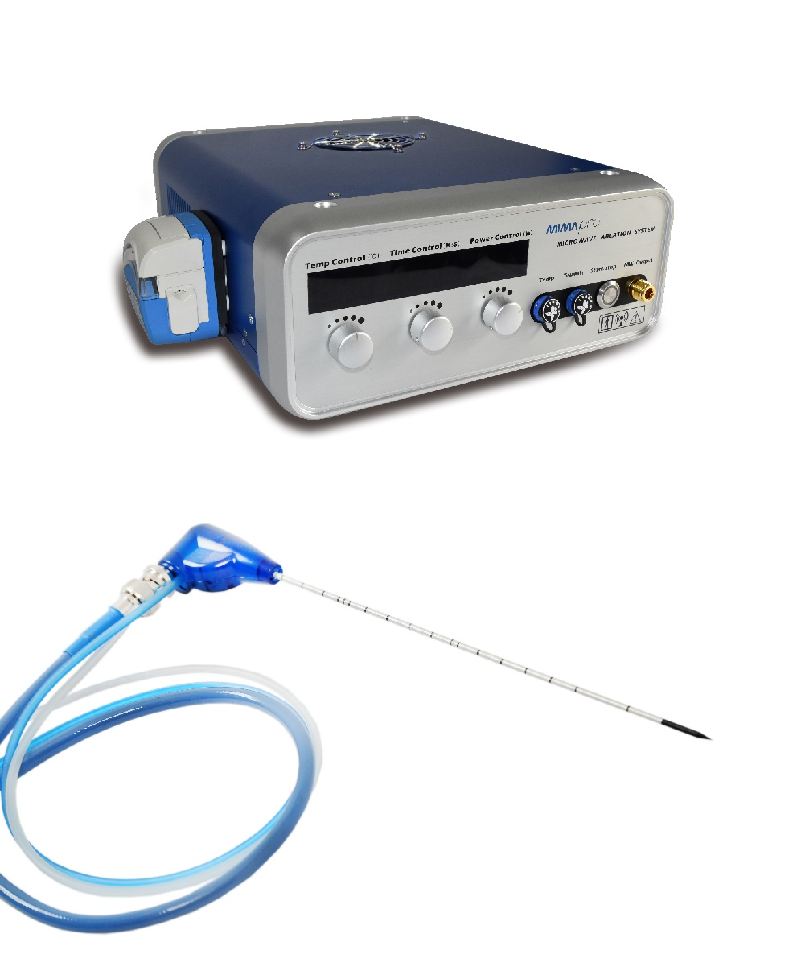

Supplement: Supplementary file 3 — Supplementary Material 3 [file 13019_2024_2677_MOESM3_ESM.png]

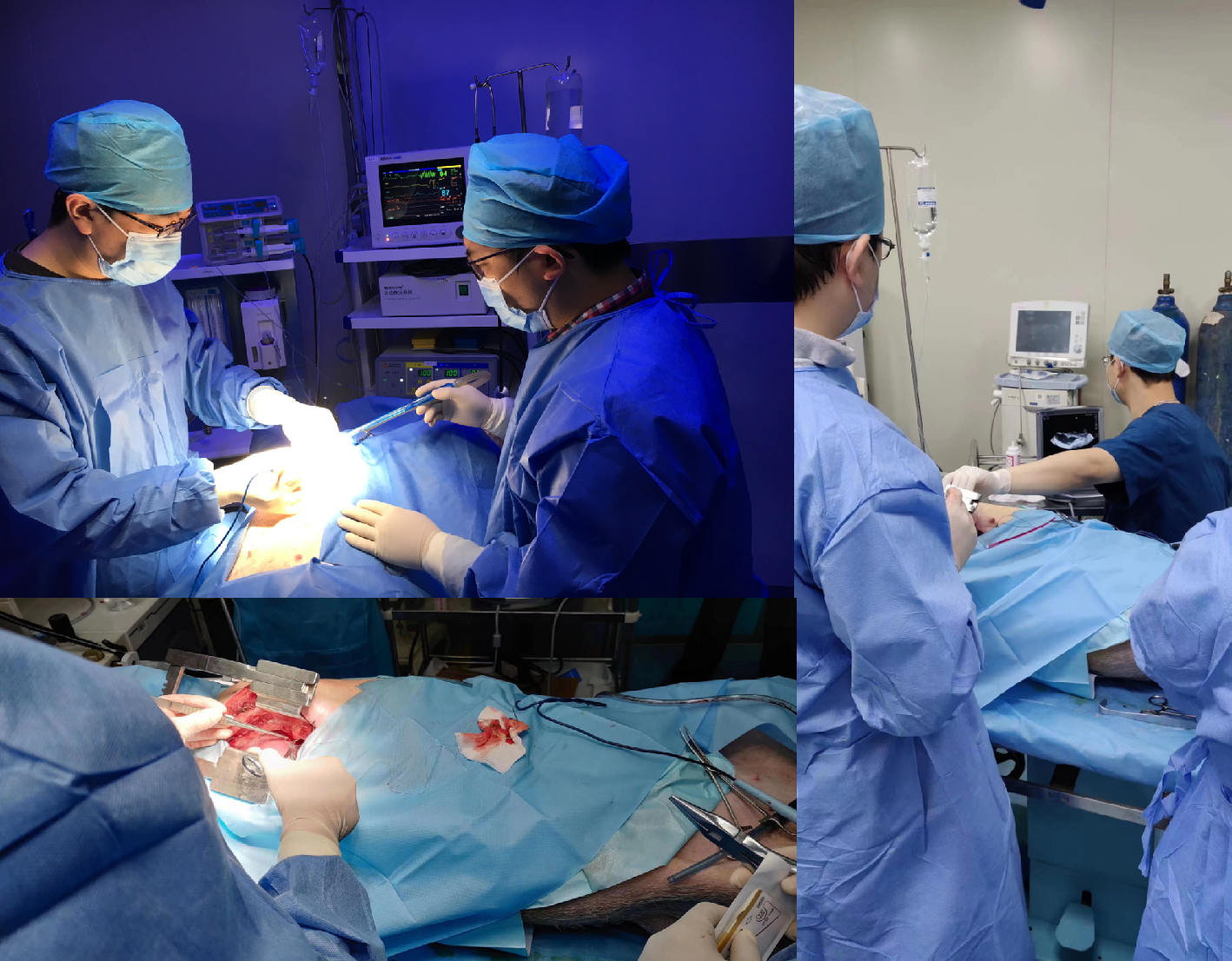

Supplement: Supplementary file 4 — Supplementary Material 4 [file 13019_2024_2677_MOESM4_ESM.png]
